# Effect of Provision of Feed and Water during Transport on the Welfare of Weaned Pigs

**DOI:** 10.3390/ani5020363

**Published:** 2015-06-04

**Authors:** Arlene Garcia, Glenna Pirner, Guilherme Picinin, Matthew May, Kimberly Guay, Brittany Backus, Mhairi Sutherland, John McGlone

**Affiliations:** 1 Department of Animal and Food Sciences, Texas Tech University, Lubbock, TX 79409, USA; E-Mails: arlene.garcia@ttu.edu (A.G.); glenna.pirner@ttu.edu (G.P.); guilherme.picinin@ttu.edu (G.P.); matthew.d.may@ttu.edu (M.M.); brittany.backus@ttu.edu (B.B.); 2 Department of Animal Science and Veterinary Technology, Tarleton State University, Stephenville, TX 76402, USA; E-Mail: guay@tarleton.edu; 3 Ruakura Research Centre, AgResearch Ltd., Hamilton 3214, New Zealand; E-Mail: mhairi.sutherland@agresearch.co.nz

**Keywords:** transportation, weaned pigs, animal welfare

## Abstract

**Simple Summary:**

Transportation is a complex stressor, which has the potential to negatively impact the health and welfare of weaned pigs. Transport duration and withdrawal from feed and water are two factors that could potentially adversely affect the welfare of pigs transported at weaning. In this study, the effect of a 32 h transport period and the provision of feed and water on the welfare of weaned pigs was investigated using a multi-disciplinary approach. Body weight decreased in weaned pigs over time and this response was exacerbated by exposing pigs to a 32 h transport period and withdrawing feed and water. The greatest changes in body weight loss were observed after 8 h of transport or weaning. Furthermore, the neutrophil to lymphocyte ratio (N:L) stress measure was elevated in pigs in response to an 8 h transport period or 8 h after weaning alone. With the exception of weaned pigs provided with feed and water, transported and weaned pigs continued to be different from control pigs until 16 h after weaning or exposure to a 16 h transport period. These findings suggest that pigs experience an acute stress response due to transport and weaning, but these two stressors do not appear to be additive. Overall, transportation had a negative effect on performance, physiology and behavior of weaned and transported pigs, especially if not provided with feed and water for more than 24 h.

**Abstract:**

Transportation is a complex stressor made up of factors including weaning itself and withdrawal from feed and water. Therefore, transportation has the potential to negatively impact the health and welfare of weaned pigs. Pigs were transported for 32 h and measures of performance, physiology, and behavior were taken to assess piglet welfare. Treatment groups included pigs not weaned or transported (CON), weaned pigs provided with feed and water (WEAN+), weaned pigs not provided with feed and water (WEAN−), weaned and transported pigs provided with feed and water (TRANS+), and weaned and transported pigs not provided with feed and water (TRANS−). Body weight loss was different among treatments (*p* < 0.01). CON pigs had a 6.5% ± 0.45% gain in body weight after 32 h. WEAN+, WEAN−, TRANS+, and TRANS− groups all had a loss in body weight of 5.9% ± 0.45%, 7.8% ± 0.45%, 6.5% ± 0.45% and 9.1% ± 0.46%, respectively. The N:L was greater in all weaned pigs at 8 h compared to CON pigs (*p* < 0.01). WEAN− and transported pigs had significantly higher N:L than CON pigs from 8 h through 16 h, however, all treatment groups were similar to CON pigs after 16 h irrespective of provision of feed and water. Blood glucose levels were lower in transported and/or weaned pigs than CON pigs after 16 h irrespective of the provision of feed and water. TRANS+ females had higher creatine kinase (CK) levels than males (*p* < 0.05). After a 16 h transport period, TRANS− pigs had higher total plasma protein (TP) levels than all other treatment groups (*p* < 0.05). Significant changes in behavior were observed during and after transportation, which could also be indicative of stress. Overall, transportation and weaning had a negative effect on performance, physiology and behavior (both during and post-weaning) of pigs, especially when feed and water was not provided. Transporting pigs without feed and water for more than 24 h was a welfare concern as indicated by changes in body weight and physiology measures of stress.

## 1. Introduction

Transportation is a complex stressor made up of many factors including fluctuating temperatures, stocking density, withdrawal from feed and water, mixing with unfamiliar pigs and motion [1]. These factors have the potential to activate the hypothalamic-pituitary-adrenal axis (HPA) in pigs. Therefore, transportation has the potential to affect the health and welfare of pigs, especially in pigs already experiencing weaning stress. We do not know if transport stress adds to weaning stress or if weaning is severe enough that adding transport has little additional negative effects. Nor can we presently state the relative stress of weaning and transport.

The practice of transporting pigs for long distances at weaning via trucks from farrowing to finishing units is becoming increasingly popular in the U.S. The main purpose for this is to reduce disease transfer and increase production [2]. However, most of the literature pertaining to the effect of transport on the stress response of pigs has been conducted on market weight pigs [3,4,5], and little is known about the welfare implications of transporting pigs at weaning. Additionally, regulations on transportation of weaned pigs are limited. In 2006, the USDA clarified that livestock conveyed by truck are also subject to be off-loaded for feed, water and rest after 28 consecutive hour of transport [6]. The 28 h law enacted in 1873 initially referred to rail transportation of cattle, sheep, swine, and other animals and was later amended to include transportation by trucks or common carriers involving confinement in a “vehicle or vessel” [6].

Length of journey is one important factor that can contribute to the overall stress experienced by pigs during transport. In market weight pigs it has been reported that the percentage of pigs that were dead on arrival at the packing plant increased as travel time increased from 30 min to 4 h [7]. A higher mortality risk has also been documented in market weight pigs transported for 30 to 90 min in the U.S. compared to transport durations of 3 h, which appeared to have a minimal effect on mortality rate [8,9]. These findings suggest that market weight pigs may experience higher levels of stress during short transport periods, which could be partially attributed to pre-transport handling. Handling can cause an acute stress response in pigs, including an increase in heart rate [10], glucose [11] and lactate concentrations [12]. Hamilton *et al.* [12] found that it took at least 2 h for physiological parameters of stress in pigs to return to baseline levels after handling. Furthermore, creatine kinase (CK), lactate dehydrogenase (LDH), adrenocorticotrophic hormone (ACTH), cortisol, insulin, triiodothyronine (T3) and thyroxine (T4) have been reported to change in response to stress [13,14,15,16] and can reflect stress coping mechanisms and the metabolic status of pigs [17]. Elevated CK levels have been associated with heart damage and have been reported to increase during a 1–2 h transport period and decrease after 4 h of transport, indicating that there may be more damage to myofibrils during the first 2 h than at 4 h of transport [17]. However, prolonged transport can cause muscle break down leading to pathological changes [17]. Therefore, it would be useful to know the optimum time for transporting pigs at weaning to reduce stress, especially as it relates to the 28 h law.

Pigs are exposed to extreme social, environmental and dietary stresses at the time of weaning which may negatively impact their immune system, performance and behavior [2,18,19]. In particular, the transition between nursing and eating solid foods may lead to a period of underfeeding, affecting growth, metabolism, and metabolic changes associated with endocrine adjustments while the animal adapts from milk to solid feed [20]. Abrupt reduction in feed intake is also known as “weaning growth check” and is more severe in some pigs than others, as some pigs keep gaining weight after they are weaned [21,22]. Pigs are commonly transported without feed and water, and weanling pigs may become dehydrated and experience muscle break down due to transport [23]. Therefore, research is needed to understand the importance of providing feed and water during transport to pigs that are consecutively experiencing weaning stress.

Increased cortisol concentrations and neutrophil to lymphocyte ratio (N:L) in weaned pigs after transport, suggest that weaning and transport conducted concurrently causes acute stress in pigs [9,23,24]. During transport, pigs may spend more time inactive, characterized by increased lying or lying/huddling, and this often increases with length of transport [19,23]. However, it is difficult to determine whether an increase in inactive behavior is due to exhaustion or habituation to the novelty of transport. Therefore when evaluating the effect of weaning and transport on pigs, it is necessary to use a multi-disciplinary approach.

It is essential for the industry to understand the impact of transport on weaned pigs, so that standards can be put into place to enhance the welfare of these animals during transport. Transportation has the potential to affect the health and welfare of pigs, especially in pigs already experiencing weaning stress. Therefore, the objective of this study was to evaluate the effect of weaning and extended transport durations (32 h) with and without feed and water on pig welfare using measures based on behavior, performance and physiology. Transport durations up to 32 h were evaluated to assess if the 28-h law in the U.S. is appropriate to maintain the welfare of pigs transported at weaning.

## 2. Experimental Design

Pigs were PIC USA genetics using the Camborough-22 sow line. All animals were fed a diet to meet or exceed NRC nutrient requirements. Feed and water were provided *ad libitum*. All animal procedures were approved by the Texas Tech University Animal Care and Use Committee.

Pigs were processed at 3 d of age (ear notched, tail docked, needle teeth clipped, and males were physically castrated). At 18 to 22 d of age (the average weaning age of pigs on commercial swine farms in the U.S.) pigs were randomly assigned to one of five treatment groups. Pigs were ear tagged 3 d prior to the beginning of the study for identification purposes. Pigs were selected by date of birth ± 2 d. Each treatment group weighed 6.0 ± 0.25 kg when selected for the study. Both gilts and barrows were evening presented in each treatment group. There were two sexes × five treatments = 10 pigs × 12 replications = 120 pigs. The study included the following treatment groups:

(1) Not weaned: pigs remained with the sow (CON)
(2) Weaned, not transported, but had access to feed and water: pigs removed from the sow and moved into nursery pens that had feed and water (WEAN+)
(3) Weaned but had no access to feed and water: pigs were removed from the sow and moved into nursery pens that did not have feed and water (WEAN−)
(4) Weaned and transported with access to feed and water: pigs were removed from the sow and transported for 32 h with access to feed and water (TRANS+)
(5) Weaned and transported without access to feed and water (as normally done): pigs were removed from the sow and transported 32 h without access to feed and water (TRANS−)

The study was conducted in the United States, near Lubbock, TX. The study was split into two sections (24 pigs/treatment × five treatments = 120 pigs/2 sections = 60 pigs/section) due to the availability of pigs and the season. The first part of the transport study was conducted in early fall and the second part in the spring during mild seasons (extreme hot and cold temperatures were avoided to prevent temperature stress). At weaning pigs were either left in the farrowing pen with the sow (CON) or placed in one of the four treatment groups. The study began at 07:00 h and ended by 16:00 h the following day. Control pigs were blood sampled by jugular venipuncture, weighed, checked for lesions or lameness every 8 h for 32 h, and placed back in the farrowing pen. The same procedures were conducted on treatment Groups 2 through 5, except that they were either placed in randomly assigned weaning pens or pens in the trailer in which they would be transported.

Pigs of the same litter in the TRANS+ and TRANS− groups were randomly placed in pens (0.76 m × 0.76 m) of two pigs (one male/one female) with wood shavings, approximately 10 cm in depth, in a gooseneck trailer (6 m × 2 m). WEAN+ and WEAN− groups were randomly placed in weaning pens (0.76 m × 0.76 m) without shavings, as they normally would be when weaned. Space allowance was determined by transport space recommendation from the TQA^®^ handbook (0.19 m^2^ per head) and the additional space (0.14 m × 0.14 m) was added to provide space for feed and water. HOBO (Onset Computer Corporation, Bourne, MA, USA) data loggers recorded temperature and humidity in the trailer. The average temperature in the trailer was 15.6 °C for the fall and 12.7 °C for the spring. Relative humidity inside the trailer ranged from 15.1% to 76.9%.

The route involved a 32 h trip, broken down into 8 h phases, in which the truck/trailer would return to the original farm site for sampling periods (approximately 15–25 min), in which pigs were weighed, bled and assessed for injuries. Feed (2.5 kg) and water consumption were determined by weighing the feed containers and by how much water was consumed from the water jugs (5 L of water were added to each jug at the beginning of the study in WEAN+ and TRANS+ pens, and water consumed was recorded every 8 h for 32 h to get the total amount of water consumed for each transport period).

Transport time continued during sampling periods, so that sampling took place at 0, 8, 16, 24 and 32 h. At the end of the trip, all pigs were placed in nursery pens (1.5 m × 1.5 m) with the same pen mate to avoid further stress caused by mixing of unfamiliar pigs.

To assess piglet welfare, measures of performance (weights, health, injury), behavior (during transport, at the farm and post-transport), and physiological changes were recorded.

*Performance:* Pigs were weighed by placing them in a plastic tub that was set on top of a digital bench scale (OHAUS, Melrose, MA, USA). The pigs were weighed before (0 h), during (at 8, 16, 24 h), immediately after (32 h), and at 7 and 14 d post study period. The percent of weight change was calculated before, during and after shipping (TRANS+ and TRANS−) or weaning (WEAN+ and WEAN−).

*Physiology:* Pigs were placed on their backs in a V-trough with their forelegs and hind legs manually restrained by trained personnel to allow access for blood collection, which took on average 2–3 min. Two milliliters of blood were collected through jugular venepuncture in BD Vacutainers^®^ (BD Company, Franklin Lakes, NJ, USA) containing 5.4 mg of K2 EDTA and an additional 2 mL were collected in BD Vacutainers^®^ without additives for blood analysis. Four milliliters in total were collected from every piglet every 8 h for 32 h. Whole blood was examined for total white blood cell (WBC) counts and differential leukocyte counts for the different white blood cell populations, hematocrit (HCT) and neutrophil to lymphocyte ratio (N:L) within 1 h after collection. Blood samples were centrifuged, and plasma and serum were collected and frozen until further analysis for cortisol concentrations and blood chemistry measures including total plasma protein (TP), albumin and creatine kinase (CK). Blood chemistry measures were conducted by Iowa State University, Department of Veterinary Pathology and were performed on the Ortho Vitros 5.1 machine (Johnson & Johnson, Auckland, New Zealand). Blood chemistry measures included glucose, total protein and creatine kinase. Cortisol concentrations were analyzed using an enzyme immunoassay kit (Enzo^®^ Life Sciences, Farmingdale, NY, USA). The resulting intra- and inter-assay CV were 6% and 10.9%, respectively. The sensitivity of the assay was 1 pg/mL.

*Behavior:* Piglet behavior was recorded for all treatment groups during and 24 h after the study. Lying, standing, sitting, drinking and eating behaviors were recorded (Table 1). Wild life cameras (Moultrie Products, Alabaster, AL, USA) were placed across from each experimental pen and were used to record behavior of TRANS+ and TRANS− treatments. Four pigs (two pens) were recorded per frame and 10 min scan samples were done for each individual pen. The cameras were motion activated and instantaneous shots were taken upon activation. Digital video recorders (DVRs) (Supercircuits^®^, Austin, TX, USA) were used to record behaviors of CON pigs in farrowing pens and weaned pigs (WEAN+ and Wean) in the nursery barn during and after the transport study. Post-transport pigs were penned with the same pen mate they had during transport. Video recordings were taken for 24 h post-transport for all groups and analyzed using 10 min scan samples in 2 h intervals.

*Statistical analysis*: The study was a Complete Randomized Design. The pen was the experimental unit, consisting of one female and one castrated male. A general linear model was used and the data were analyzed using analysis of variance procedures in SAS. The statistical model included the effects of sex, treatment, pen within treatment, time, season and all possible interactions. Pen within treatment was used as the model’s error term. No interaction between seasons was observed. An F-protected Least Significant Difference (LSD) test was also used within SAS. Behavior was analyzed in a general linear model using the analysis of variance procedure in SAS. The behavior observations were done in 10 min scan samples in 2 h intervals. All data were tested for homogenous variances and normal distributions. All data were analyzed using SAS 9.3 General Linear Models procedure (SAS, SAS Inst. Inc., Cary, NC, USA, 2010).

**Table 1:** Ethogram with description of behaviors observed during the 32 h study period and 24 h post-weaning and/or transport.

Behavior	Definition
Lying	Animal is recumbent, flat on its side, or ventrally
Standing	Animal is upright on all fours, legs are extended
Sitting	Resting on the caudal part of the body with the forelimbs extended
Drinking	Animal placing its mouth on the water nipple and consuming water
Eating/Nursing	Animal placing its mouth in the feed container and consuming feed or latching on to the sows’ nipple.

## 3. Results and Discussion

### 3.1. Weight

**Figure 1:**
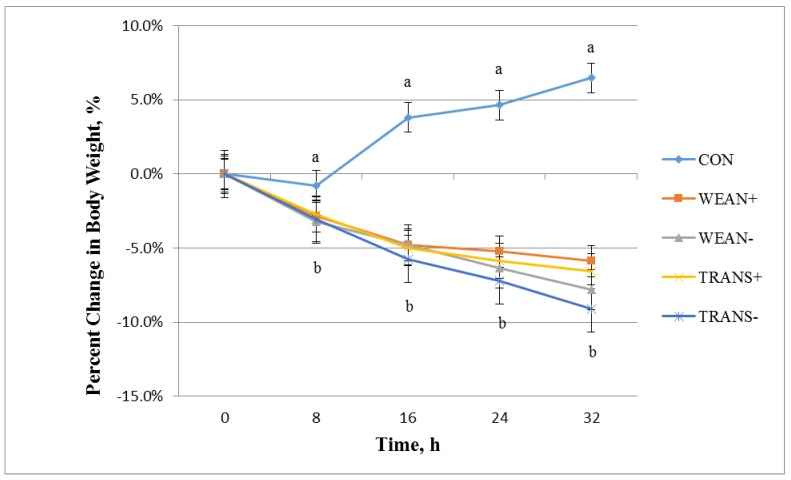
Least squares means ± SEM for percent change in body weight over 32 h for pigs weaned and/or transported with or without feed and water, and for non-weaned non-transported pigs (CON) (*p* < 0.01). Treatments: not weaned, not transported (CON; n = 24); weaned, not transported, provided feed and water (WEAN+; n = 24); weaned, not transported and not provided feed and water (WEAN−; n = 24); weaned and transported, provided feed and water (TRANS+; n = 24); and weaned and transported, not provided feed and water (TRANS−; n = 24). CON is different from all other treatments at *p* < 0.05 and TRANS− is different from WEAN+ at *p* < 0.05.

The treatment by time interaction was significant for percent change in body weights (*p* < 0.01). All treatments were different from the CON group at 8 h (Figure 1). TRANS− pigs differed in percent loss in body weight from WEAN+ and TRANS+ pigs at 24 h (*p* < 0.05), but did not differ from the WEAN− group. At 32 h, TRANS− pigs differed in percent loss in body weight from all treatment groups (*p* < 0.05). WEAN+ pigs had a similar loss in body weight as TRANS+ pigs, but differed from WEAN− and TRANS− pigs (*p* < 0.05). The additional loss in percent body weight recorded in TRANS− and WEAN− pigs was likely due to the lack of feed and water. These findings have also been documented in slaughter weight pigs that have been transported and fasted for 25 to 48 h [5,11,24,25,26,27,28,29,30,31].

### 3.2. Physiology

#### 3.2.1. Neutrophil to Lymphocyte Ratio Interaction

A significant treatment by time interaction for N:L was observed (*p* < 0.01). All treatment groups were different from CON at 8 h (Table 2). By 16 h only WEAN+ pigs were similar to CON pigs, and all other treatments significantly differed from WEAN+ and CON pigs (*p* < 0.05). Sutherland *et al.* [23,24] found that concurrent weaning and transport caused an increase in the cortisol and N:L response in weaned pigs. The results from the present study and Sutherland *et al.* [23,24] suggest that pigs experience acute stress due to transport and/or weaning. Furthermore, it is well documented that weaning alone (the lack of maternal warmth, milk and pheromones, social and environmental changes) is a considerable stressor [19] and in conjunction with transport and handling can result in physiological and behavioral changes. However, stress caused by these two stressors (weaning and transport) does not appear to be additive.

From 24 to 32 h, there were no differences (*p* > 0.05) in N:L among treatments. The similarity in the N:L among treatments after 24 h could be due to adaptation of the pigs to transport and/or weaning.

**Table 2:** Least squares means for neutrophil to lymphocyte ratio (N:L) for treatment by time interaction (*p* < 0.01; Pooled SE = 0.09). Treatments: not weaned, not transported (CON; n = 24); weaned, not transported, provided feed and water (WEAN+; n = 24); weaned, not transported, and not provided feed and water (WEAN−; n = 24); weaned and transported, provided feed and water (TRANS+; n = 24); and weaned and transported, not provided feed and water (TRANS−; n = 24).

Treatment	0 h	8 h	16 h	24 h	32 h
**CON **	0.59	0.64 ^a^	0.70 ^a^	0.69	0.86
**WEAN+ **	0.77	1.10 ^b^	0.84 ^a^	0.61	0.76
**WEAN− **	0.67	1.16 ^b^	1.10 ^b^	0.87	0.82
**TRANS+**	0.62	1.0b	1.00 ^b^	0.84	1.06
**TRANS− **	0.73	1.0b	1.10 ^b^	0.69	0.64

^a,b^ Means with different superscripts within time periods differ at *p* < 0.05.

#### 3.2.2. Blood Glucose Interaction

A treatment by time interaction for blood glucose was observed (*p* < 0.01). All treatment groups differed from the CON at 8 h (*p* < 0.05; Table 3). Blood glucose concentrations increased (*p* < 0.05) in CON pigs from 0 to 8 h, which may have been due to the amount of stress they were undergoing, as activation of sympathetic hormones can cause an increase in blood glucose. Additionally, CON pigs had more milk availability since their littermates had been weaned. At 16 h, TRANS+ and TRANS− groups had similar blood glucose levels to CON pigs, while WEAN+ and WEAN− pigs had lower blood glucose levels than CON pigs (*p* < 0.05). There was a significant difference between the WEAN+, WEAN− and TRANS+ and TRANS− groups by 16 h; blood glucose levels of transported pigs were higher than weaned pigs. Furthermore, blood glucose levels remained higher in transported compared to weaned pigs throughout the remainder of the study. Blood glucose levels of both transported and weaned pigs remained lower (*p* < 0.05) than CON pigs after 16 h of transport.

**Table 3:** Least squares means for treatment by time interaction for blood glucose (*p* < 0.01; transported, Pooled SE = 3.6) Treatments: not weaned, not transported (CON; n = 24); weaned, not provided feed and water (WEAN+; n = 24); weaned, not transported, and not provided feed and water (WEAN−; n = 24); weaned and transported, provided feed and water (TRANS+; n = 24); and weaned and transported, not provided feed and water (TRANS−; n = 24).

Treatment	0 h	8 h	16 h	24 h	32 h
**CON**	122.45	135.01 ^a^	121.89 ^a^	130.45 ^a^	124.16 ^a^
**WEAN+**	123.15	116.12 ^b^	104.96 ^b^	82.24 ^b^	87.12 ^b^
**WEAN−**	124.05	114.38 ^b^	104.66 ^b^	86.82 ^b^	83.85 ^b^
**TRANS+**	117.03	121.42 ^b^	114.53 ^a^	103.08 ^b^	103.36 ^b^
**TRANS−**	116.24	122.19 ^b^	119.30 ^a^	107.13 ^b^	102.96 ^b^

^a,b^ Means with different superscripts within time periods differ at *p* < 0.05.

The difference in blood glucose levels between weaned and transported pigs could potentially be attributed to an increase in the sympathoadrenal responses stimulated by aspects of transport, such as noise during transport, rough driving, bumpy roads, or any combination of these that transported pigs were exposed to in comparison to the quiet and warm environments in which the weaned and CON pigs were. At 24 h and up to 32 h of being weaned or transported, all treatment groups had significantly lower blood glucose levels than the CON group. Blood glucose levels decreased over time for weaned and transported pigs even if provided with feed, indicating that the amount of feed consumed, if any, was not enough to increase or sustain their blood glucose levels. Pigs were not provided with creep feed in farrowing, thus, they had not learned or adapted to eating solid feed prior to the study. This may be one reason why they did not consume as much feed as they needed to main blood glucose levels. Higher levels of blood glucose in response to 8 h of transport have been documented in finishing pigs [32]. It was reported that this response was due to a shorter fasting period compared to pigs exposed to a 16 h transport period [32]. Blood glucose levels may be maintained during short-term fasting, partially due to hepatic gluconeogenesis [33]. However, stress induced hyperglycemia has been documented to be suppressed in 24 h fasting pigs [34]. In the current study, greater differences in glucose concentration between treatments after 16 h were possibly due to the combination of longer fasting periods and the addition of stress caused by transport. Additionally, stimulation of hepatic gluconeogenesis in young pigs may be different than that of older pigs. During the suckling period gluconeogenesis is activated because milk does not meet the glucose requirement of the piglet and body fat accretion is primarily derived from milk [35,36]. The switch from high fat, low carbohydrate liquid milk to low-fat, high carbohydrate solid feed causes gluconeogenesis to become transient, lipogenesis to become marginal and activates lipolysis [37].

#### 3.2.3. Creatine Kinase Interaction

A sex by treatment interaction for CK was observed (*p* < 0.01). TRANS+ female pigs had higher (*p* < 0.05) CK levels than TRANS+ males, 1687.3 ± 253.2 IU/L and 543.8 ± 370.4 IU/L, respectively (Table 4). There were no sex differences among the other treatment groups.

**Table 4:** Least squares means ± SEM for treatment by sex interaction for creatine kinase (CK; *p* < 0.01). Treatments: not weaned, not transported (CON; n = 24); weaned, not transported, provided feed and water (WEAN+; n = 24); weaned, not transported, and not provided feed and water (WEAN−; n = 24); weaned and transported, provided feed and water (TRANS+; n = 24); and weaned and transported, not provided feed and water (TRANS−; n = 24).

Treatment	Male	Female	SE
**CON**	1153.60	2002.70	453.30
**WEAN+**	1749.09	1042.71	332.45
**WEAN−**	647.05	1392.65	350.05
**TRANS+**	543.84 ^a^	1687.34 ^b^	311.80
**TRANS−**	547.02	1083.38	325.50

^a,b^ Means with different superscripts within each treatment differ at *p* < 0.05.

CK is released from muscle fibers into the circulation in response to muscular activity and/or tissue damage [14,38,39] and can indicate physical fatigue [40]. Elevated CK levels after transport have been documented in weaned pigs immediately after transport [9,24,41]. Sex has been shown to affect stress responses of pigs during transport, however, most studies have shown this response in older animals transported to slaughter [42,43]. Previous studies have shown higher CK levels in gilts than barrows [44] possibly indicating that females may be more stress-susceptible than males or differences may also be due to castration of males.

#### 3.2.4. Total Plasma Protein Interaction

A significant TP treatment by time interaction was found among treatment groups (*p* < 0.01). TP levels for TRANS− pigs differed (*p* < 0.05) from all other treatment groups by 16 h and remained different until 32 h (Table 5). By 32 h, all treatment groups had higher TP levels than CON pigs, including the WEAN+ and TRANS+ groups which had remained similar to the CON group from 0 to 24 h. The significant increase in TP in the WEAN+ and TRANS+ groups compared to the CON group may have been due to the amount of water the pigs were drinking. Although water was provided, TRANS+ and WEAN+ pigs may not have consumed sufficient water to maintain TP values similar to those found in CON pigs. Increased TP levels may suggest that TRANS and WEAN pigs were experiencing dehydration as a result of feed and water deprivation and transport. Previous studies have reported lower TP levels in pigs after transport durations as short as 60 min [23]. Although, TP, albumin concentrations and hematocrit have been reported to increase after transport [9,23,24], in the current study, albumin and hematocrit values were not significantly different among treatment groups, suggesting that the level of dehydration experienced by the pigs in this study was not sufficient to affect other indicators of dehydration.

**Table 5:** Least squares means for total protein and time interaction over a 32 h transportation period (*p* < 0.01; Pooled SE = 0.07). Treatments: not weaned, not transported (CON; n = 24); weaned, not transported, provided feed and water (WEAN+; n = 24); weaned, not transported, and not provided feed and water (WEAN−; n = 24); weaned and transported, provided feed and water (TRANS+; n = 24); and weaned and transported, not provided feed and water (TRANS−; n = 24).

Treatment	0 h	8 h	16 h	24 h	32 h
**CON**	5.33 ^a^	5.17 ^a^	5.18 ^a^	5.09 ^a^	4.97 ^a^
**WEAN+**	5.14 ^b^	5.22 ^a^	5.28 ^a^	5.17 ^a^	5.22 ^b^
**WEAN−**	5.06 ^b^	5.17 ^a^	5.35 ^a^	5.16 ^a^	5.21 ^b^
**TRANS+**	5.12 ^b^	5.16 ^a^	5.27 ^a^	5.17 ^a^	5.18 ^b^
**TRANS−**	5.15 ^b^	5.29 ^b^	5.50 ^b^	5.40 ^b^	5.44 ^b^

^a,b^ Means with different superscripts within time periods differ at *p* < 0.05.

#### 3.2.5. Cortisol

Plasma cortisol concentrations were similar among treatment groups (*p* > 0.05). Typically elevated cortisol concentrations and increased N:L levels are found in response to stress and have been reported to be elevated in weaned pigs after transport [9,23,24]. It was apparent that all animals, including the CON animals, were stressed due to repeated handling and blood sampling in this study. Elevated cortisol concentrations in response to the stress caused by repeated handling and blood sampling may have masked the effect of the stress caused by weaning and transport. Blood collection is an invasive procedure and a limitation when assessing stress in animals. In future studies it may be beneficial to use non-invasive measures of stress, such as measuring glucocorticoid concentrations in the feces or saliva of pigs. However, in the present study other indicators of stress (e.g., N:L) were elevated in pigs experiencing weaning and transport compared to CON, suggesting that these stressors were still perceived as being greater than repeated handling in pigs.

### 3.3. Behavior

#### 3.3.1. During Transport 

Overall, the CON group spent 65% of their time lying during the 32 h study period. TRANS+, TRANS−, WEAN+ and WEAN− pigs all spent over 75% of their time lying (TRANS+: 77%; TRANS: 81%; WEAN+: 81%; WEAN−: 87%).

The CON group and WEAN− treatment group pigs spent 11% of time standing during the 32 h study. TRANS−, TRANS+ and WEAN+ treatment groups stood respectively 14%, 16%, and 17% of the time during the 32 h study period.

**Figure 2:**
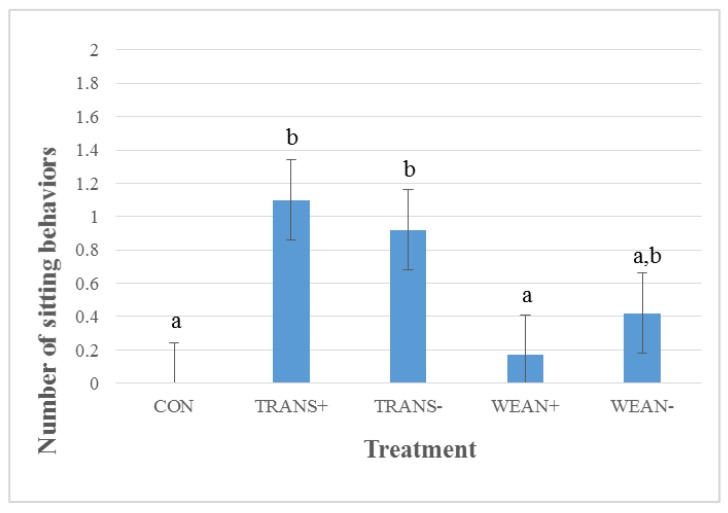
Least squares means ± SEM for number of sitting behaviors for treatment groups during a 32 h transport study (*p* < 0.01). Treatments: not weaned, not transported (CON; n = 24); weaned, not transported, provided feed and water (WEAN+; n = 24); weaned, not transported, and not provided feed and water (WEAN−; n = 24); weaned and transported, provided feed and water (TRANS+; n = 24); and weaned and transported, not provided feed and water (TRANS−; n = 24). ^a,b^ Means with different superscripts differ at *p* < 0.05.

There was a significant treatment effect for sitting behavior (*p* < 0.01). Both TRANS+ and TRANS− treatment groups sat significantly more (*p* < 0.05) than the CON group (Figure 2). The WEAN+ treatment group spent a similar amount of time sitting as the CON group. WEAN− pigs spent a similar amount of time sitting as CON, TRANS+ or TRANS− treatment groups. The CON group did not spend any time sitting during the study. The WEAN+ treatment group spent 1% of their time sitting, while WEAN−, TRANS− and TRANS+ treatment groups spent respectively 2%, 4% and 5% of their time sitting.

Lying and standing were the most prevalent behaviors observed in early-weaned experimentally transported pigs during transport [29]. Furthermore, lying and standing rates of 75.6% and 21.6%, respectively, have been reported in weaned pigs during transport [29]. High levels of resting after weaning, could influence lying levels during transport [45]. In addition, as transport time increases, lying behaviors also tend to increase [23,29]. Sitting is a behavior that has been identified as a potential stress indicator [46] and is more common during the first 12 h of transport (2.8%) than during the second 12 h (0.3%) [29]. However, increased standing or sitting behavior may also be an indication of competition for space or instability due to too much space [30]. Increased resting and decreased sitting behaviors later in transport may indicate that pigs have become habituated to some of the elements of transport [30].

A significant treatment effect on feeding behavior was observed (*p* < 0.01). CON pigs ate more (nursing) than both TRANS+ and WEAN+ pigs (*p* < 0.05). TRANS+ and WEAN+ pigs did not differ in their eating behaviors. CON pigs spent 18% of their time eating during the 32 h study. WEAN+ pigs spent 0.43% and TRANS+ pigs spent 0.35% of their time eating during the 32 h study period. Occasionally equipment failure occurred resulting in periods of missed data collection. However, these results still show that weaning markedly disrupts eating behavior in pigs.

It is typical to see reduced feeding behavior during the first days post-weaning. Feeding has been infrequently observed (0.5%) on the first day of weaning whether pigs were transported or not [30]. The results from the present study are consistent with other reported studies [45,47,48]. Furthermore, space allowance, season, and transport duration can increase resting, drinking, and feeding behaviors post-transport [2], possibly indicating that weaned pigs experience dehydration, hunger and fatigue in response to transport [29,41].

**Figure 3:**
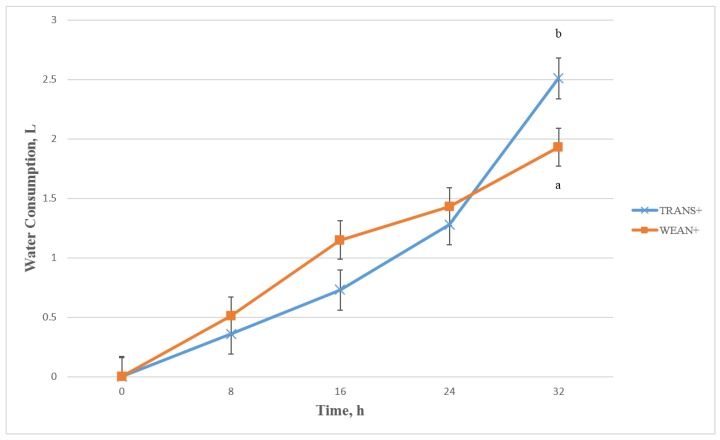
Least squares means ± SEM for time by treatment interaction for water disappearance during a 32 h transport study (*p* < 0.01). Treatments: weaned, not transported, provided feed and water (WEAN+; n = 24); weaned and transported, provided feed and water (TRANS+; n = 24). ^a,b^ Means with different superscripts within time periods differ at *p* < 0.05.

There was a significant treatment by time interaction for water consumption during the 32 h study period (*p* < 0.01). It is likely that water is spilled and wasted among weaned pigs. Water disappearance may be correlated with water consumption. Water disappearance did not differ between TRANS+ and WEAN+ treatment groups from 0 h to 24 h (Figure 3). However, after 24 h the TRANS+ treatment group drank significantly more water than the WEAN+ treatment group (*p* < 0.05), 2.51 ± 0.17 L and 1.93 ± 0.16 L by 32 h, respectively. Thus, water consumption increased in a linear fashion as time of transport increased.

#### 3.3.2. Post-Treatment Behavior

Overall, CON pigs spent 81% of their time lying during the 24 h post-treatment period. CON pigs were weaned at the end of the 32 h study period, therefore lying behavior may have increased in these animals as a response to the weaning experience. During the experimental period, CON pigs were repeatedly handled for blood sampling, therefore the increase in lying behavior may be associated with these animals recovering from this repeated stress.

The WEAN+ treatment group spent 80% of the time lying, almost the same amount of time as the CON group. The WEAN− treatment group spent 75% of their time lying. The TRANS+ and TRANS− treatment groups spent 74% and 71% of their time lying, respectively.

**Figure 4:**
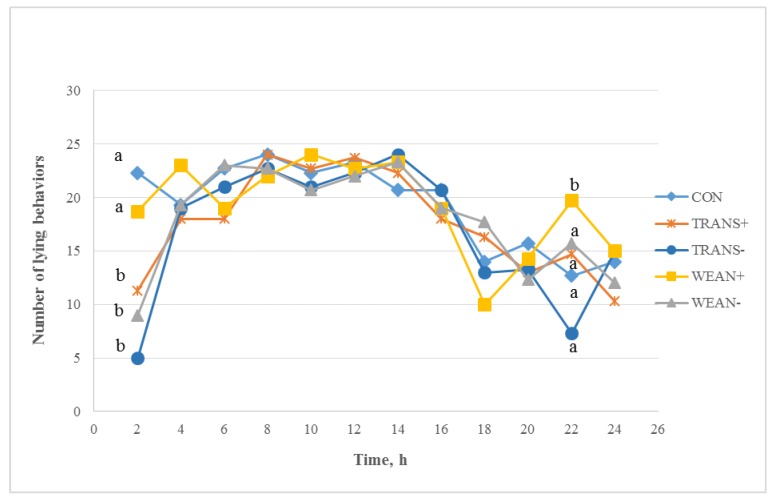
Least squares means ± SEM for time by treatment interaction for lying behaviors in 2 h intervals post-treatment (*p* < 0.05; pooled SE = 2.0). Treatments: not weaned, not transported (CON; n = 24); weaned, not transported, provided feed and water (WEAN+; n = 24); weaned, not transported, and not provided feed and water (WEAN−; n = 24); weaned and transported, provided feed and water (TRANS+; n = 24); and weaned and transported, not provided feed and water (TRANS−; n = 24). ^a,b^ Means with different superscripts within time differ at *p* < 0.05.

The treatment by time interaction for lying behavior during the 24 h post-treatment period was significant (*p* < 0.01). Lying behaviors were different at 2 and 22 h post-treatment. At 2 h post-treatment, lying times were similar between WEAN+ pigs and CON pigs, and the time spent lying was higher than for the other treatment groups (Figure 4). TRANS+, TRANS−, and WEAN− pigs differed significantly in lying behaviors compared to CON pigs at 2 h post-treatment. TRANS+, TRANS− and WEAN− pigs spent less time lying than CON and WEAN+ pigs (*p* < 0.05). At 22 h post-treatment all treatment groups displayed similar lying times to CON pigs except for WEAN+ pigs. WEAN+ pigs spent more time lying than all other groups 22 h post-treatment (*p* < 0.05). From 4 to 20 h and at 32 h post-treatment lying behaviors were similar among treatments. Space allowance, season and transport duration can affect the behavioral response of weaned pigs, both during and post-transport [9,24,29].

Only the TRANS− treatment group displayed sitting behaviors post-treatment, but the incidence of this behavior was low (1%). After transport, standing frequency has been seen to drop and lying behavior frequency has been seen to increase [30], possibly due to fatigue. Sitting behaviors have previously been attributed to muscle fatigue [25] and have been reported as an indication of stress [46].

#### 3.3.3. Feed and Water Consumption during and after Transportation

Post-treatment feed and water consumption was recorded at 7 and 14 d, but no significant differences were found among treatments. Post-treatment weights were taken at 1, 7, and 14 d. There was also no significant difference in weight among treatment groups. These findings indicate that pigs overcame the stressful event of weaning and transportation without feed and water similarly to recently weaned pigs that had access to the sow during the study (*i.e.*, CON pigs).

## 4. Conclusions

Literature pertaining to the effect of transportation on the welfare of weaned pigs is limited [5,23,29,49,50,51,52,53,54] and comparison of studies is sometimes difficult due to the variation in methodology, pig age/weight and densities studied [30]. Stress during transportation is inevitable and is generally aggravated by loading and unloading, vibration, coping with a new environment, restricted space, mixing of conspecifics, lack of ventilation, and deprivation of feed and water [55].

In the current study, body weight performance was reduced with duration of transport and lack of feed and water and was significantly different than for CON pigs by 8 h. Weaning caused a loss of body weight, but weaning without feed and water caused pigs to lose more weight than those provided with feed and water. Pigs that were weaned and transported with feed and water lost a similar amount of weight as those weaned with feed and water. Pigs transported without feed and water lost more weight than animals that were weaned without feed and water. Therefore, weaned and transported pigs benefit from feed and water starting at 24 h after weaning and transport.

Transport and weaning had negative impacts on physiology of the pigs in comparison to CON pigs. Based on the current findings both weaned and transported pigs experienced an acute increase in N:L by 8 h. However, N:L in pigs that were weaned and provided with feed and water returned to values similar to those of CON by 16 h, whereas all other pigs had similar values to CON pigs by 24 h. Other physiological measures such as blood glucose and total plasma protein worsened as transport time increased. Thus, both weaning and duration of transport with or without feed and water negatively affect pig well-being and the 28 h law may need to be modified. Providing feed and water on journeys may help to reduce some of the detrimental effects caused by long distance transport. In total, the data support a recommendation of providing feed and water if transport is greater than 24 h based on measures of body weight, physiology and behavior.

Physiological measures were also seen to increase in response to prolonged stress. Although cortisol levels were not significant among treatment groups, studies have shown that supplementing pigs with magnesium mica (MM) can aid in reducing blood cortisol levels as well as catecholamine concentrations [56,57,58] and result in calmer pigs after long distance transportation [59]. Further research in reference to the use of physiological measure of stress are needed. Some physiological measures that merit further study include, acute phase proteins (APP), including pig major acute phase proteins (Pig-MAP), haptoglobin, serum amyloid A (SAA), and C-reactive protein (CRP), which have recently been suggested as a good means of measuring animal welfare [60,61] and are said to increase in response to inflammation due to tissue damage, infections, immunological disorders, or stress [62,63].

Transportation is important from both animal welfare and economic perspectives. Further studies are needed to determine whether feed or water alone can help maintain body weight, to improve weaned pig welfare during transport.
